# SIRT1 haploinsufficiency recapitulates age-associated subfertility through sperm α-tubulin hyperacetylation

**DOI:** 10.1186/s13062-025-00710-2

**Published:** 2026-03-25

**Authors:** María Iniesta-Cuerda, Iveta Valentova, Jiří Moravec, František Liška, Milena Králíčková, Dario Krapf, Jan Nevoral

**Affiliations:** 1 Biomedical Center, Faculty of Medicine in Pilsen, Charles University, alej Svobody 76, Pilsen, 323 00 Czech Republic; 2 Pronatal Sanatorium, Na Dlouhé Mezi 12/4, 147 00 Prague 4, Prague, Czech Republic; 3 First Faculty of Medicine, Charles University, Kateřinská 1660/32, Prague, 121 08 Czech Republic; 4 Department of Histology and Embryology, Faculty of Medicine in Pilsen, Charles University, Alej Svobody 1655/76, Pilsen, 323 00 Czech Republic; 5 Laboratory of Cell Signal Transduction Networks, Instituto de Biología Molecular y Celular de Rosario (IBR), CONICET-UNR, Blvd. 27 de febrero 210 bis, Rosario, S2000EZP Argentina; 6 Laboratory of Reproductive Medicine, Faculty of Biochemical and Pharmaceutical Sciences, National University of Rosario, Suipacha 531, Rosario, S2002LRK Argentina

**Keywords:** SIRT1, Aging, Acetylation, Sperm

## Abstract

Advanced paternal age compromises male fertility and correlates to a decline of the deacetylase SIRT1 activity, a central regulator of germline homeostasis and chromatin dynamics. Acetylation imbalance has been pointed out as a driver of testicular aging. We therefore asked whether SIRT1 insufficiency reproduces aging-associated shifts in the acetylation landscape and how these might propagate to sperm function, fertilization capacity and embryo development. To address this, we combined acetylomic profiling of *Sirt1*^+^/^−^ and naturally aged mice with functional assays of sperm quality and in vitro fertilization (IVF). Both *Sirt1*^*+*^*/*^*−*^ and aged wild type (WT) testes shared a distinct acetylation signature absent in young WT controls, including nuclear regulators (ZNF638, MORC4), proteins involved in sperm structure and motility (Rootletin, Kinectin, CFAP58), and phosphoinositide signaling mediators such as PLCη1, which regulate intracellular Ca^2 +^ release. Conversely, 22 proteins displayed acetylation exclusively in WT controls but were absent in *Sirt1*^*+*^*/*^*−*^ and aged testes, encompassing modules related to flagellar organization (CEP170, CEP350, AKAP13), meiotic control (ANAPC7, PDS5B, ESCO1, CENPE), signaling and metabolism (SHIP1, GAK, ABCD4), chromatin regulation (BRD4, MAGEB4), and testicular architecture (MFAP2). This differential acetylomic profile persisted in mature sperm, with *Sirt1*^+^/^−^ males showing elevated acLys levels and, more specifically, midpiece-restricted α-tubulin hyperacetylation, a pattern particularly shared with aged cohorts. Notably, this tubulin hyperacetylation remained after capacitation and correlated with mitochondrial dysfunction, elevated reactive oxygen species, reduced acrosome responsiveness, and diminished fertilization capacity. IVF assays further revealed decreased cleavage and blastocyst developmental rates, indicating defective paternal support of early embryogenesis despite preserved blastocyst quality. Together, these findings indicate that testicular SIRT1 contributes to germline acetylation patterns and that midpiece-restricted α-tubulin hyperacetylation is a shared feature of SIRT1 insufficiency and natural aging, correlating with the mitochondrial dysfunction and impaired sperm performance. Overall, our work broadens current understanding by integrating acetylomic and functional evidence within a model that reflects the physiological, age-related reduction of SIRT1.

**Supplementary Information:**

The online version contains supplementary material available at 10.1186/s13062-025-00710-2.

## Introduction

The trend towards higher parental age in couples is increasingly prevalent, and this demographic shift is exerting pressure on reproductive capabilities, often leading to subfertility [1]. In men, aging results in a progressive decline in various physiological functions and negatively impacts on testicular function [2] and sperm quality [3–5], challenging fertilization. Additional aspects contributing to paternal aging related subfertility have been associated with increased risk of pregnancy loss, birth defects, offspring diseases [6, 7], and even stillbirth [8]. Consequently, there is an immediate need to comprehend the significance of advanced paternal age and its potential adverse impact not only on sperm capacity but also on embryogenesis and offspring health.

In this context, various proteins have been identified to undergo age-related changes, and a group of NAD^+^ -dependent histone deacetylases, known as Sirtuins, has emerged as crucial protective factors against aging [9–11]. SIRT1 deficit has been associated with the precipitation of reproductive aging in animals that are supposed to be in their active sexual period. Conversely, 3-month-old mice lacking SIRT1 (*Sirt1*^−^/^−^) experience severe testicular dysfunction akin to aging [12–14]. As a result, sperm quality is significantly affected, showing an aging-like increase in abnormal forms, immotile spermatozoa, and DNA abnormalities, reducing fertilization success even in in vitro fertilization (IVF) assays [13–18]. Based on our recent findings, testicular SIRT1 insufficiency and natural aging converge on a shared molecular basis: on one hand, the loss of spermatogenic proteins and factors involved in cellular protection, repair, and homeostasis that are uniquely present in young WT males; on the other hand, the selective enrichment of factors mediating oxidative stress responses and mitochondrial compensation [19]. Altogether, this scenario delineates a testicular environment chronically engaged in stress adaptation.

Beyond its roles in stress resistance and longevity, SIRT1 fine-tunes the male germline proteome through targeted deacetylation events that govern protein stability, activity, and localization [18, 20–22]. In the testis, SIRT1 deacetylation of FOXO1 supports germline maintenance and antioxidant defenses, while reduced deacetylation of PGC-1α impairs mitochondrial biogenesis and redox balance, leading to elevated ROS and deficient sperm metabolism [16, 20]. Thus, SIRT1 supports a molecular program that preserves germ-cell development and equips spermatozoa for their functional requirements. Since mature sperm lack transcriptional and translational activity [23], proteins required for fertilization must be synthesized, and many of them modified during testicular development, epididymal maturation and capacitation [24]. Reduced SIRT1 activity during testicular development may imprint the germline proteome with altered acetylation patterns, which could affect the molecular context needed for efficient fertilization. Clinical evidence supports this view, as reduced α-tubulin acetylation, a constitutive modification established during sperm development and essential for flagellar stability [25], has been frequently observed in subfertile patients and correlates with impaired sperm motility [26]. Consistent with these findings, we observed that testicular SIRT1 insufficiency yields sperm with a diminished capacity to undergo hyperactivation during capacitation and an excessive accumulation of mitochondrial ROS [27], potentially representing the functional manifestation of underlying defects in acetylation-dependent regulation.

Building on these premises, this study set out to determine whether the subfertility associated with reduced testicular SIRT1, as occurs during natural aging, is accompanied by reproducible changes in the testicular acetylation landscape and whether these alterations extend to mature sperm, particularly those shared with naturally aged males. Using a *Sirt1*^+^/^−^ model that mimics the age-associated decline rather than the complete loss of SIRT1, we generated a qualitative, reproducible overview of acetylated peptides and integrated it with functional assessments of sperm performance, capacitation-associated remodeling, and IVF outcomes. This combined approach expands previous work on SIRT1 by revealing molecular and functional features shared between SIRT1 insufficiency and aging, including a consistent midpiece hyperacetylation of α-tubulin within a broader context of impaired sperm physiology.

## Material and methods

### Chemicals and reagents

All chemicals and reagents used for the experiments were purchased in Merck Millipore or Thermo Fischer Scientific, unless otherwise stated.

### Animals

Mice used in the experiments were euthanized by cervical dislocation, in accordance with the ActNo0.246/1992, on the Protection of Animals against Cruelty, under supervision of the Animal Welfare Advisory Committee at the Charles University, Faculty of Medicine in Pilsen, and approved by the Animal Welfare Advisory Committee at the Ministry of Education, Youth and Sports of the Czech Republic (Ethics approval number: MSMT-33798/2021–4). Euthanasia was performed sequentially, with CO₂ narcosis followed by cervical dislocation. Anesthesia is not required for any of the experiments described. This work has been reported in accordance with the ARRIVE 2.0 guidelines. All mice used in the experiments were housed in cages at 21 ± 2 °C, light/dark cycles of 12:12 with *ad libitum* access to food and water. The *Sirt1*^±^ experimental males were generated as described earlier [27]. Briefly, *Sirt1* conditional knock out (Zp3-Cre:*Sirt1*^loxPloxP^) females, with excised exon 5–7 (*Sirt1*^Δ5–7^) in oocytes, were mated with wild-type (WT) C57Bl/6N males. F1 males were used for back-cross with WT females, producing *Sirt1*^±^ experimental males and *Sirt1*^+/+^ siblings that were used as WT control. The 3.5-months-old WT and *Sirt1*^±^ males were used in our experiments. For the evaluation of the aging effects on male reproductive performance, WT animals reaching 12 months were considered as aged (Old WT).

### Sample collection of testicular tissue and sperm isolation

Testes and spermatozoa were obtained from *Sirt1*^+^/^−^ males, their WT (*Sirt1*^+^/^+^) littermates and aged WT counterparts. Following euthanasia, testes were excised and the tunica albuginea was removed, then testicular extracts were prepared using RIPA buffer. For sperm recovery, epididymides were dissected into 0.5 mL of Human Tubular fluid (HTF; 101.6 mM NaCl, 4.7 mM KCl, 0.4 mM KH₂PO₄, 2.0 mM CaCl₂·2 H₂O, 1.2 mM MgSO₄·7 H₂O, 25.0 mM NaHCO₃, and 40.6 mM Na lactate), allowing sperm to swim out for 15 min. Both sperm samples and testicular tissues were subsequently processed according to the downstream analyses performed.

### Proteome profiling of testes by LCMS

Testicular lysates prepared in RIPA buffer were subjected to in-solution digestion with trypsin overnight at 37 °C. All samples were processed freshly and in parallel under identical conditions to minimize technical variation and avoid artifactual modifications. Peptide mixtures were then analyzed using a nanoLC system coupled to a trapped ion mobility quadrupole time-of-flight mass spectrometer (timsTOF Pro, Bruker, USA). For each experimental group (minimum of three animals), samples were run in four technical replicates. All groups were processed side-by-side under the same protocol to ensure comparability. Downstream analyses included only those proteins consistently detected in at least three technical and all three independent biological replicates. Tandem MS spectra were interrogated with lysine acetylation and N-terminal acetylation set as variable modifications. A detailed description of the LC-MS workflow is provided in the Supplementary methods S1.

### Electrophoresis and western blot

Testicular and sperm lysates were prepared in RIPA buffer and subsequently combined with Laemmli loading buffer before electrophoretic separation and Western blotting, following established protocols [28]. Prior to antibody incubation, membranes were blocked and then probed sequentially with the appropriate primary and secondary antibodies. Detection of immunoreactive bands was achieved using the ECL Select Western Blotting Detection Reagent (GE Healthcare Life Sciences, UK), and signal acquisition was performed on a ChemiDoc™ MP Imaging System (Bio-Rad, France). Membrane images were analyzed with ImageLab software (v6.0.1, Bio-Rad, France), and band intensity was quantified as adjusted volume. For normalization, α-tubulin was employed as a loading control in testicular lysates, whereas stain-free total protein detection was used for sperm samples. A full list of antibodies and detailed procedures is provided in the Supplementary Methods S1.

### Dot-blot of acetylated proteins

Amersham Protran Premium 0.45 µm nitrocellulose membranes (GE Healthcare, #10600008) were cut into 8 × 3 cm rectangles and gridded with a soft pencil into thirty-two 1 × 1 cm squares. Onto each square, 1 µL of sample prepared in RIPA buffer was applied and allowed to dry. Three biological replicates were evaluated corresponding to three different males of each experimental group. Membranes were washed twice for 1 min with TTBS (0.1% Tween-20 in Tris-buffered saline) and blocked for 1 h with 3% Blotting-Grade Blocker (Bio-Rad, #1706404) in TTBS. After blocking, membranes were washed three times (3 × 1 min) with TTBS and incubated overnight at 4 °C with anti-acetyl-lysine antibody (Abcam, #ab80178) diluted 1:1,000 in TTBS. The following day, membranes were washed three times (3 × 1 min) with TTBS and incubated for 2 h at room temperature with HRP-conjugated secondary antibody (anti-Rabbit IgG H&L, Abcam, #ab6721) diluted 1:10,000 in TTBS. After five additional washes (5 × 1 min) with TTBS, signals were developed using Clarity Western ECL substrate (Bio-Rad, #1705060) according to the manufacturer’s instructions. Images were acquired with a ChemiDoc Imaging System (Bio-Rad) using ImageLab 4.1 software (Chemi-Hi Sense settings). All estimations were performed in triplicate. Image processing and spot intensity detection were performed using ImageLab 4.1 software. Spots were selected with the round volume tool applying local background subtraction and a constant area. Intensity values were exported to Excel for statistical analysis, normalized to the sum of all spot intensities across the membrane, and triplicates were tested for outliers using the Grubbs test. Validated values were averaged and normalized to the BCA protein concentration.

### Evaluation of sperm parameters

Functional attributes of spermatozoa were examined under basal conditions (directly after epididymal isolation), following capacitation, and after induction of the acrosome reaction (AR) in vitro. Flow cytometry analyses were performed using at least three biological replicates per group, with each replicate corresponding to an individual animal. Capacitation was achieved by incubating sperm in HTF supplemented with 0.4% (w/v) BSA (mHTF) for 1 h at 37 °C, and AR was induced by adding progesterone (20 µM) to capacitated sperm for 10 min at 37 °C. For the evaluation of acrosome and membrane integrity, mitochondrial activity, reactive oxygen species levels (ROS) and membrane lipoperoxidation, spermatozoa were stained with corresponding fluorochromes (PNA-FITC, Mitotracker deep Red, 2´,7´-dichlorodihydrofluorescein diacetate (H2DCFDA) and BODIPY^®^ 581/591, respectively), and both signal intensities and percentage of positive/negative spermatozoa were recorded. In all these evaluations, the viable sperm population was distinguished by applying the Live/Dead Fixable kit (L/D) according to manufacturer´s instructions, or propidium iodide (PI) for 5 min at room temperature (RT). PI or Live/Dead dyes were used as appropriate to avoid spectral overlap and to match fixation requirements. Spermatozoa were stained through their incubation in the corresponding media added with the specific fluorochrome for 30 min at 37 °C, except when using PI and PNA-FITC that 4 min and RT were applied. For the evaluation of acrosome integrity slightly modified published protocols were used [29]. Briefly, spermatozoa were simultaneously fixed and permeabilized after L/D staining with PBS supplemented with paraformaldehyde (PFA; 4% (w/v)), Triton-X 100 (0.1% (v/v)) and Tween-20 (0.2% (v/v)) at room temperature (RT) for 1 h and PNA-FITC was applied as above mentioned. A FACSVerse Flow Cytometer (BD Biosciences) controlled with BD FACSuite was used and for each sample, 10,000 events were recorded. Excitation of PNA-FITC, H2DCFDA and BODIPY^®^ 581/591was performed with a blue laser (488 nm); Mitotracker deep Red was excited with far red laser (647); green fluorescence was detected with a 537/32 BP filter; red fluorescence was detected with a 700/54 BP filter. Data were analyzed using WEASEL Ver. 3 (WEHI) and a gating approach to select the sperm population was applied as previously described [27]. Briefly, sperm were identified on FSC vs SSC, viability gates were applied using L/D or PI signal.

### Immunocytochemistry

Proteins in spermatozoa and embryos were analyzed using adapted immunocytochemistry (ICC) protocols [30]. To exclude dead cells, sperm were first labeled with L/D. Both sperm and embryos were subsequently fixed, permeabilized, and blocked in PBS containing paraformaldehyde (4% w/v), Triton X-100 (0.1% v/v), Tween-20 (0.2% v/v), and BSA (5% w/v), prior to incubation with primary antibodies (1:1,000) (mouse anti–acetyl-lysine (acLys; ADI-KAP-TF120-E, Enzo Life Sciences), rabbit anti–acetyl-α-tubulin (acTub; ab24610, Abcam, UK), mouse anti–phosphotyrosine (pY; #05–321, Sigma Aldrich), mouse anti-Cdx2 (#bs1620R, Bioss), and rabbit anti–Oct4 (#ab217250, Abcam). Visualization was achieved using Alexa Fluor 488– and 647–conjugated secondary antibodies. Negative controls were performed by omitting the primary antibody. Samples were counterstained with DAPI and mounted in Vectashield, and images were acquired with an Olympus I×83 spinning-disk confocal microscope. For the evaluation of pY patterns, 100 sperm per sample were scored. Classification followed predefined criteria based on the regional distribution of fluorescence: (i) post-acrosomal, (ii) acrosomal, (iii) midpiece-enriched, (iv) principal piece-enriched pattern. Scoring was performed by a trained evaluator blinded to experimental groups. Because assessments were conducted by a single scorer, inter-rater agreement statistics do not apply; however, blinding and standardized criteria were used to minimize observer bias and ensure reproducibility. Flow cytometry was used to quantify fluorescence intensities and the proportion of marker-positive viable sperm, as well as to classify distinct pY patterns. In blastocysts, Cdx2- and Oct4-positive cells were enumerated to evaluate trophectoderm (TE) and inner cell mass (ICM) allocation, respectively.

### In vitro fertilization assay

For IVF assays, a portion of spermatozoa capacitated in mHTF was used. Capacitation was carried out for at least 1 h at 37 °C under 5% CO₂ in a humidified atmosphere. In parallel, cumulus–oocyte complexes (COCs) were retrieved from hormonally primed ICR females (8–12 weeks old, PMSG–hCG) as previously described [27]. Capacitated sperm were then co-incubated with COCs at a final concentration of 1 × 10^6^ sperm/mL for 5.5 h in mHTF medium. Presumptive zygotes were subsequently cultured in EmbryoMax-KSOM medium supplemented with 0.1% BSA until the blastocyst stage. Developmental progression was monitored by recording fertilization at 6–8 h post-insemination (hpi), cleavage at 24 hpi, and blastocyst and hatching rates at day 4 post-insemination. Data were expressed as percentages of zygotes (number of zygotes related to MII oocytes) cleaved embryos (number of 2-cell embryos relative to of zygotes), blastocysts (number of expanded blastocysts relative to 2-cell embryos), and hatched blastocysts (hatched blastocysts relative to expanded blastocysts). MI oocytes were excluded from analysis. Blastocysts were fixed as described above and subjected to ICC.

### Statistics

Data analysis was performed using GraphPad Prism 8 (GraphPad Software Inc., San Diego, CA, USA). Normality of distribution was assessed with the Shapiro–Wilk test. For datasets following a normal distribution, comparisons were made using paired Student’s *t*-test or analysis of variance (ANOVA), followed by Tukey’s post-hoc test when appropriate. For non-normally distributed data, the Kruskal–Wallis test was applied, with Dunn’s test used for multiple comparisons. Pearson correlations were calculated using population-level sperm values to assess relationships between acetylation markers and functional parameters, noting that the reduced sample size warrants cautious interpretation. Given the relatively small sample size, these correlations should be interpreted with caution, as coefficients may be less stable under these conditions. A threshold of *p* < 0.05 was considered statistically significant. Results are reported as means for normally distributed data and medians for non-normally distributed data.

## Results

### SIRT1 insufficiency drives distinct aging-linked acetylome signatures

**Fig. 1:**
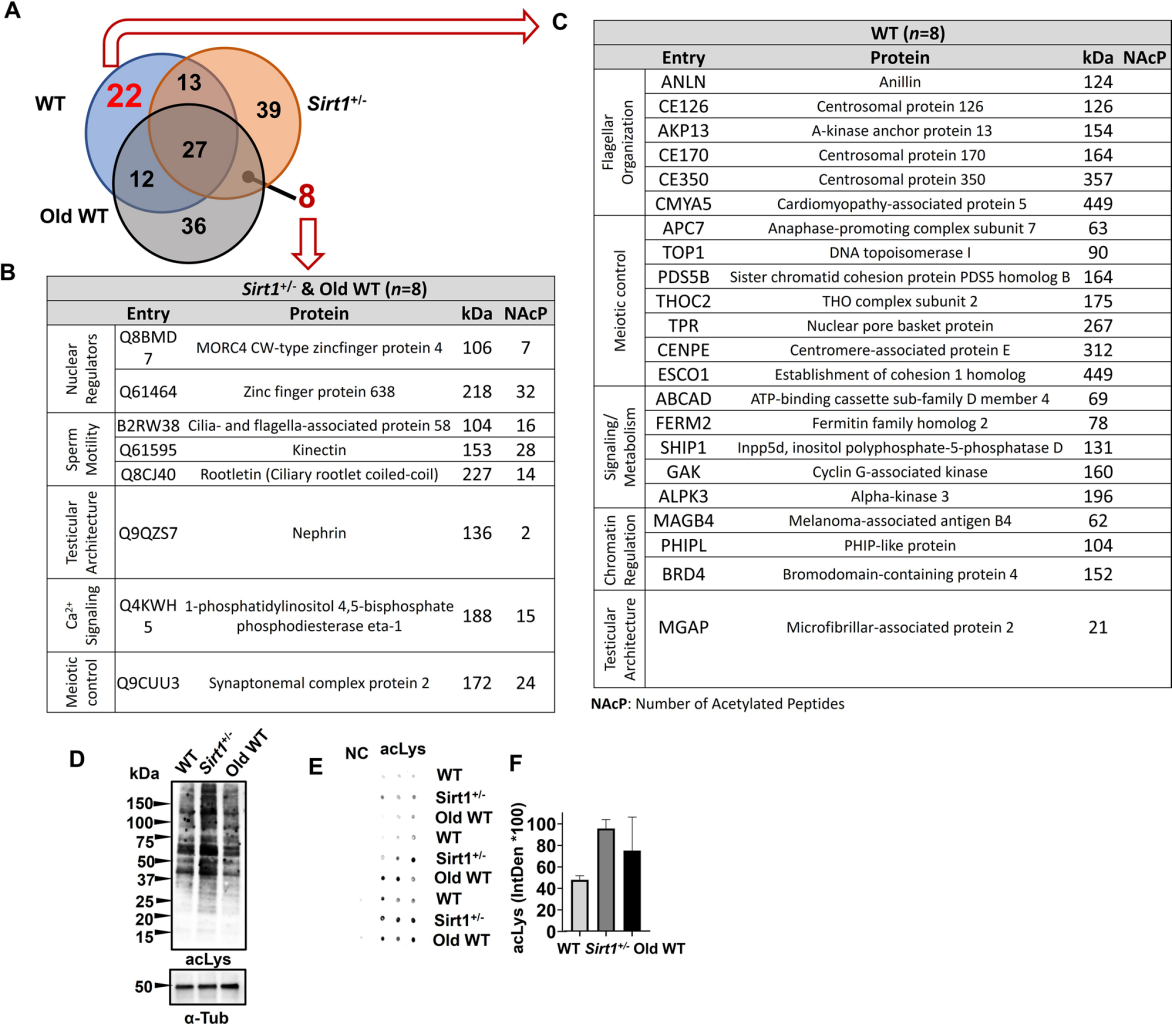
*Sirt1*^+^/^−^ testes show aging-like acetylomic signatures. (**A**) venn diagram depicts (**B**) the overlap of acetylated proteins between *Sirt1*^+^/^−^ and aged WT testes, as well as (**C**) the subset lost in both but exclusively present in WT. Representative list of proteins uniquely acetylated in (**B**) *Sirt1*^+^/^−^ and aged WT testes versus those present in WT, suggesting convergent molecular alterations driven by insuficient SIRT1 activity. Proteins were clustered according to panther classification system, with the number of acetylated peptides (NAcP) indicated. Notably, shared proteins across both models encompassed factors with direct implications for nuclear regulators, sperm motility, testicular architecture, Ca2+ signaling and meiotic control, while lost proteins included regulators of flagellar organization, meiosis, metabolism, chromatin, and testicular architecture. (**D**) Western blot analysis of acetylated lysine (acLys) showed different patterns of global protein acetylation. α-tubulin served as a loading control. (**E**) dot blot illustrates the global acetylation landscape of testicular proteins (acLys; *n* = 3), and (**F**) its integrated density (IntDen) quantification, where bars represent mean ± s.e.m. NC: negative control with primary antibody omitted. (*n* = 3)

SIRT1 haploinsufficiency has been shown to induce a testicular phenotype that mimics aging, primarily due to the loss of proteins involved in cellular maintenance during aging and the emergence of pro-senescence factors [19]. These whole-proteome alterations may be a consequence of persistent hyperacetylation in upstream regulators, which remains evident in both *Sirt1*^+^/^−^ and aged WT males as a direct result of reduced SIRT1 deacetylase activity. To test this hypothesis, a comprehensive evaluation of the acetylation landscape was developed by mass spectrometry which identified acetylated peptides and linked them to their corresponding proteins. Strikingly, this analysis uncovered eight proteins acetylated exclusively in both *Sirt1*^+^/^−^ and aged WT testes but absent in young WT controls (Fig. 1A–B). Among them, nuclear regulators such as Zinc finger protein 638 and MORC4 CW-type zincfinger protein 4 were found to be hyperacetylated, supporting the notion that their altered acetylation could act as an upstream effector mechanism, driving the observed proteomic alterations and contributing to the cascade of events leading to reproductive aging. Moreover, we identified key proteins involved in sperm motility, such as Rootletin, Kinectin, and Cilia- and flagella-associated protein 58, displaying distinct acetylation patterns in *Sirt1*^+^/^−^ and aged WT testes compared with WT controls. Likewise, components of the phospholipase C family, including PLCη1 (1-phosphatidylinositol 4,5-bisphosphate phosphodiesterase eta-1), which transduces phosphoinositide signals to elicit intracellular Ca^2 +^ release, exhibited altered acetylation. Conversely, 22 proteins were acetylated exclusively in WT controls but not in either *Sirt1*^+^/^−^ or aged WT testes (Fig. 1A–C). Proteins relevant to motility and flagellar organization, such as CEP170, CEP350, Anillin, AKAP13, and CMYA5; together with proteins essential for cell division and spermatogenesis, including ANAPC7, PDS5B, ESCO1, CENPE, TOP1, THOC2, and TPR; as well as factors involved in signaling and metabolism (SHIP1, Kindlin-2, GAK, ABCD4), nuclear and chromatin regulation (BRD4, Mageb4, PHIP-like), and testicular architecture (Mfap2), displayed an acetylation pattern exclusively detected in WT testes but absent in both *Sirt1*^+^/^−^ and aged WT animals. The complete acetylome dataset is provided in the Supplementary Data S2. Having identified specific substrates with altered acetylation, we also examined whether these local changes were reflected in global acetylation profiles. For this purpose, testicular lysates were analyzed by Western blotting with anti–acetyl-lysine antibodies, which showed modest variations in band intensity across samples (Fig. 1D). Consistently, dot-blot measurements revealed a slight upward trend in global acetylation in *Sirt1*^+^/^−^ and Old WT testes, although this tendency was not statistically significant (Fig. 1E–F).

### Persistent acetylation changes in spermatozoa highlights α-tubulin as one target of testicular SIRT1 insufficiency and aging

**Fig. 2:**
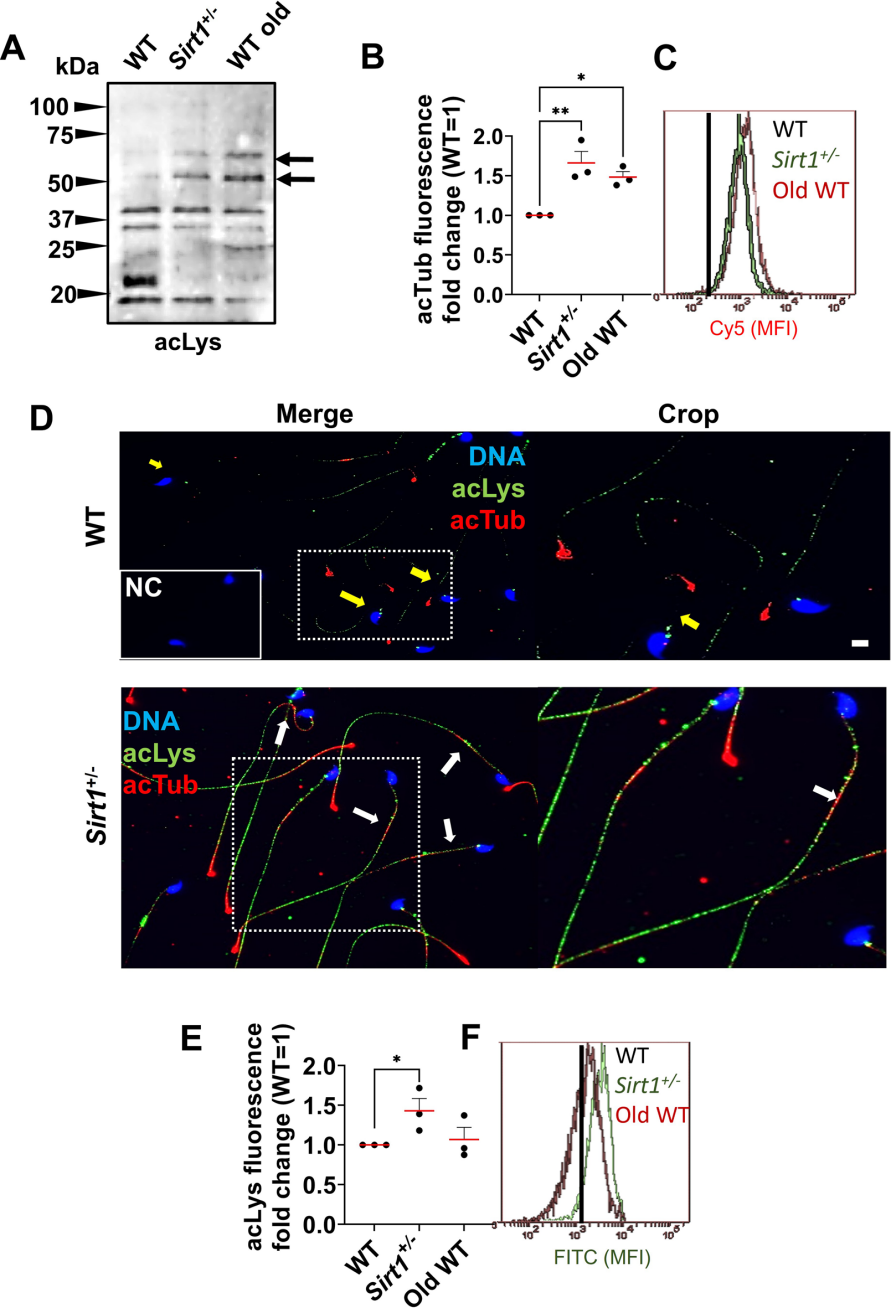
Constitutive acetylome of old WT and *Sirt1*^*±*^ spermatozoa. Evaluation of the whole-acetylated proteome analysis of sperm by (**A**) Western blot using anti-acetylated substrates in lysine 40 antibody (acLys). Stain-free total protein detection was used as a loading control for sperm lysates. Arrow marks candidate hyperacetylated substrates in old WT and *Sirt1*^+^/^−^ spermatozoa. Acetylated α-tubulin (acTub) was chosen for evaluation and (**B**) flow cytometry revealed its hyperacetylation. acTub fluorescence is shown relative to that of WT (at a value of 1). (**C**) Histogram of Cy5 mean fluorescence intensity (MFI) reflecting acTub levels in viable sperm, with the WT reference shown in black and SIRT1^+^/^−^ and aged WT populations in green and red, respectively. (**D**) Representative micrographs of the inmunocitochemistry of acLys and acTub in spermatozoa. DNA was counterstained with DAPI to delineate sperm nuclei. Arrows indicate absence (yellow) and accumulation (white) of acTub at the mid piece of the flagellum. Negative controls (NC) were included by omitting the primary antibody, confirming specificity of the immunostaining. (**D**) evaluation of the whole-acetylated proteome analysis of sperm by flow cytometry. acLys results are presented as Fold change from the WT control (assigned a value of 1) are based on fluorescence intensity measurements obtained. (**F**) Histogram of FITC MFI, reflecting acLys levels in the viable sperm population, with the WT reference shown in black and *Sirt1*^+^/^−^ and aged WT populations in green and red, respectively. Dots represent individual biological replicates and lines show median; (* *p*≤0.05, ** *p*≤0.01). Scale bar: 20 µm

Having established that SIRT1 insufficiency coincides with alterations in the testicular acetylome, we next examined whether similar changes were present in mature sperm and associated with their reduced fertilization performance in vivo (Iniesta-Cuerda et al., manuscript under final revision). Consistently, sperm isolated from *Sirt1*^+^/^−^ and aged WT males exhibited an altered acetylation pattern compared to WT controls. Western blot analyses revealed enhanced acetyl-lysine signals at approximately 55 and 75 kDa in both *Sirt1*^+^/^−^ and aged sperm, suggesting that specific proteins within these molecular-weight ranges may undergo differential acetylation (Fig. 2A). Considering that acTub (≈55 kDa) is a major structural component of the sperm flagellum [31], that it displayed a distinct acetyl-lysine signal within the molecular-weight range detected by our pan-Kac blots, and that altered acTub levels have been consistently linked to defects in sperm architecture and motility [26], including those previously observed in our *Sirt1*^+^/^−^ model during capacitation [27], we selected acTub as a biologically meaningful and technically robust target for evaluation. Accordingly, flow cytometry-based quantification confirmed greater accumulation of acTub in viable *Sirt1*^+^/^−^ and aged sperm (*p* < 0.05; Fig. 2B–C). Imaging of immunostained sperm localized acTub predominantly to the flagellar midpiece (Fig. 2D). Importantly, when global lysine acetylation was quantified by flow cytometry, no significant differences were observed in aged WT sperm (*p* > 0.05), in contrast to the clear increase detected in *Sirt1*^+^/^−^ samples (*p* < 0.05; Fig. 2E). These observations suggest that the effects of natural aging may be less detectable when acetylation is examined globally, whereas SIRT1 insufficiency reveals changes at the level of individual substrates, consistent with the testicular phenotype we observed. In this context, the consistent hyperacetylation of α-tubulin in both *Sirt1*^+^/^−^ and aged samples points to a shared alteration that aligns the two conditions.

### Sperm α-tubulin hyperacetylation is linked to age-related mitochondria deficiency and concurrent sperm dysfunction

**Fig. 3:**
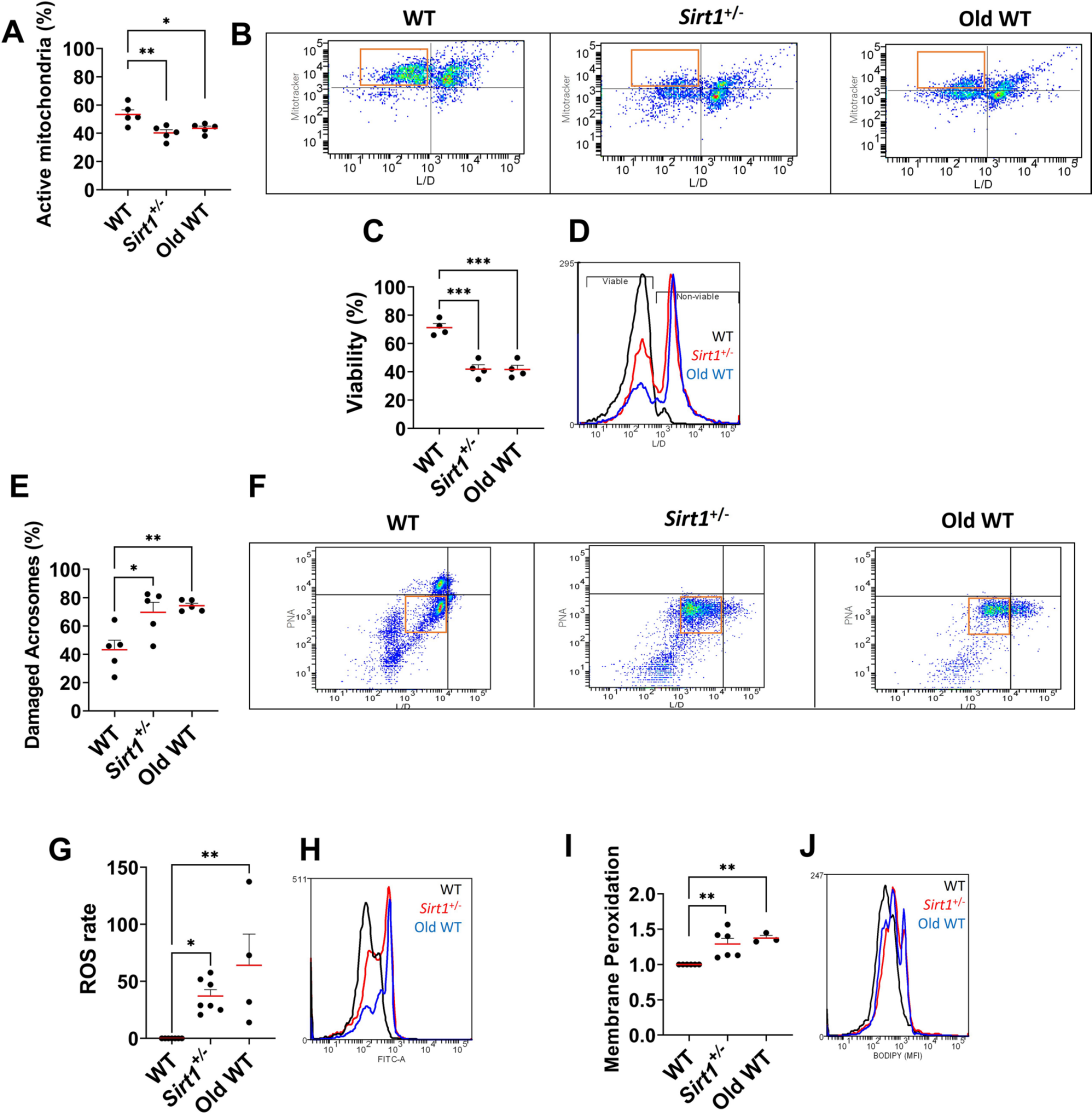
Spermatozoa exhibiting α-tubulin hyperacetylation display mitochondrial dysfunction accompanied by oxidative stress markers, reflecting a decline in overall functional quality. (**A**) mitochondrial activity, (**B**) sperm viability and (**C**) acrosome integrity was evaluated by flow cytometry. In addition, markers of oxidative stress ((**D**) ROS accumulation and (**E**) membrane lipoperoxidation) are shown as additional signs of sperm degeneration. All accompanying micrographs correspond to representative flow-cytometry dot-plots or histograms. ROS levels and membrane peroxidation of wt sperm were considered as reference and then compared with those of the experimental groups. Dots represent individual biological replicates and lines show median. (* *p*≤0.05, ** *p*≤0.01, *** *p*≤0.001)

With α-tubulin hyperacetylation appearing as a shared feature of SIRT1 insufficiency and aging, we next examined its functional consequences. Because mitochondria coil around the flagellar midpiece, which is organized on a tubulin-based axoneme (microtubules) [32], we assessed mitochondrial activity to examine whether the observed tubulin hyperacetylation is associated with changes in mitochondrial function. This analysis revealed a marked impairment in mitochondrial activity (Fig. 3A). Given the central role of mitochondria in sperm physiology, we next assessed key functional parameters by flow cytometry, including membrane integrity, acrosome status and redox balance. Consistently, both aging and *Sirt1*^+^/^−^ sperm exhibited signs of progressive deterioration, including reduced viability, compromised acrosome integrity, elevated ROS levels, and increased membrane peroxidation compared to WT sperm (Fig. 3B–E, *p* < 0.05). To further examine this relationship, correlation analyses showed that hyperacetylation of α-tubulin is strongly associated with impaired sperm quality. Significant correlations were observed between acTub levels and mitochondrial dysfunction, compromised acrosome integrity, and membrane peroxidation (Table 1; *p* < 0.05). In line with these observations, the overall decline in sperm function was significantly correlated with mitochondrial impairment (Table 1). Taken together, these observations suggest that testicular SIRT1 may influence the acetylated state of sperm α-tubulin, and that reduced SIRT1 activity might be associated with the rapid functional decline seen after sperm isolation.

**Table 1:** Correlations among mitochondrial activity, acTub and other sperm attributes

Sperm	Viability	Acrosome damage	Active mitochondria	ROS rate	Membrane Peroxidation
acTub	−0.593 *P* = 0.10	**0.763** *P*** = 0.02**	**−0.780** *P*** = 0.02**	0.517 *P* = 0.16	**0.914** *P*** = 0.002**
Active mitochondria	**0.817** *P*** = 0.01**	-**0.767** *P*** = 0.021**	-	**−0.814** *P*** = 0.012**	**−0.729** *P*** = 0.034**

### SIRT1 insufficiency disrupts sperm capacitation dynamics, reduces acrosome responsiveness thereby leading to subfertility

**Fig. 4:**
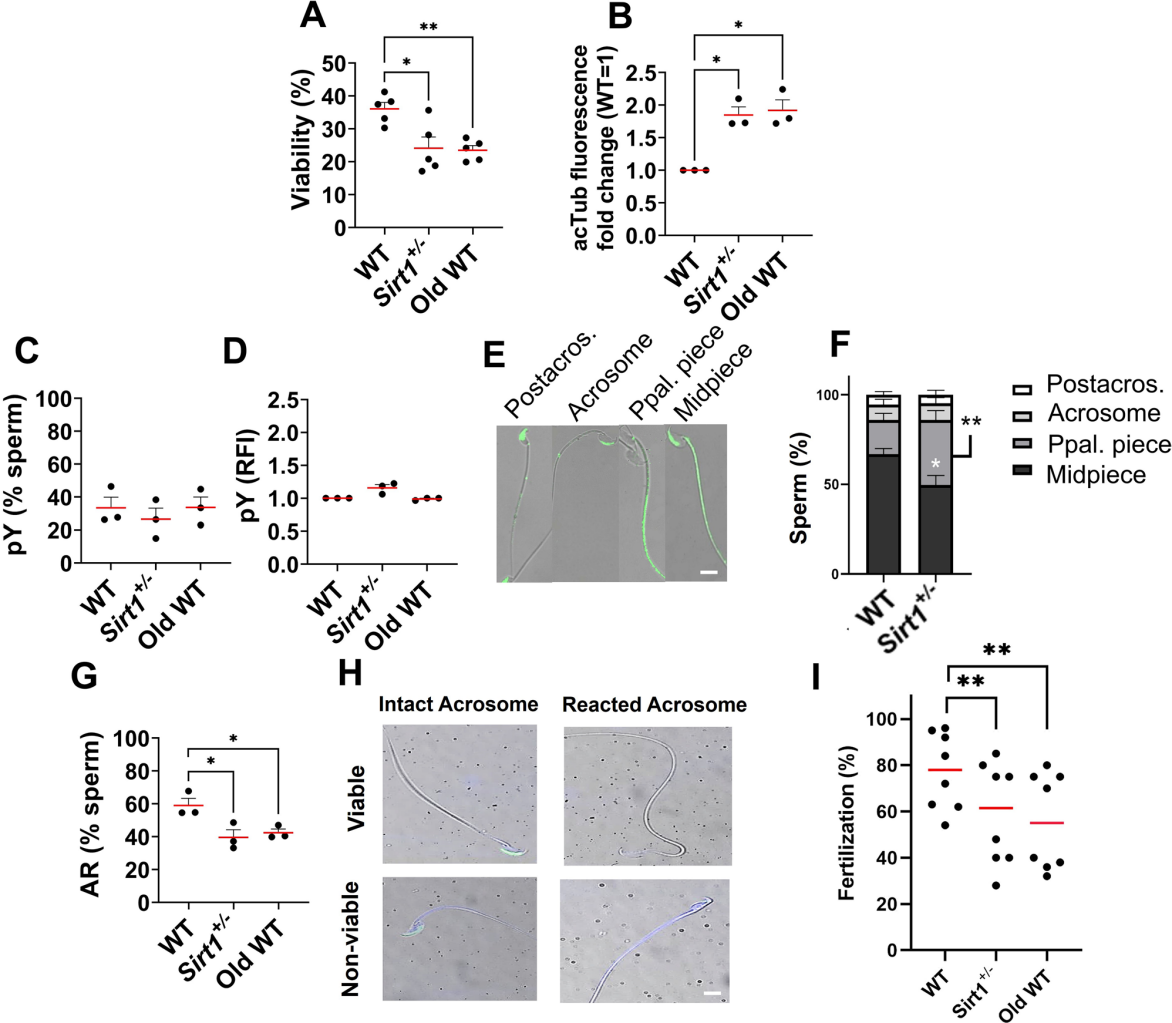
Aged WT and *Sirt1*^+^/^−^ spermatozoa that remain viable are less competent to undergo capacitation-associated modifications. (**A**) acetylated α-tubulin (acTub) remained elevated in the subset of sperm that survived 1 h under capacitating conditions compared with WT. (**B**) viability after capacitation was assessed by flow cytometry. As a marker of capacitation, protein tyrosine phosphorylation (pY) was analyzed: (**C**) the percentage of pY^+^ viable sperm and (**D**) the relative fluorescence intensity (RFI) of pY^+^ viable sperm are shown, with RFI expressed as Fold change relative to WT (WT = 1). (**E**) immunocytochemistry revealed four distinct pY localization patterns, postacrosomal region, acrosomal cap, principal piece, and midpiece, which were quantified, and (**F**) results are expressed as percentages. (**G**) progesterone-induced acrosome reaction (ar) was evaluated, and the proportion of viable sperm undergoing ar is shown. (**H**) Representative pictures of the ar evaluations. Only fully capacitated sperm can achieve fertilization, so an in vitro fertilization assay was performed, (**I**) with fertilization competence expressed as the percentage of zygotes relative to MII oocytes. Dots represent individual biological replicates and lines show median. Stacked columns represent mean ± s.e.m. (* *p*≤0.05, ** *p*≤0.01). Scale bar: 10 µm

Since capacitation is required for fertilization competence, we next evaluated sperm viability after 1 h under capacitating conditions. After incubation, sperm viability was reduced in both *Sirt1*^+^/^−^ and aged WT groups compared with WT controls (Fig. 4A). Within the surviving population, these groups also exhibited increased α-tubulin hyperacetylation relative to controls (Fig. 4B). To further investigate capacitation in these mouse models, we evaluated pY levels, a well-established marker of capacitation [33, 34]. As shown in Fig. 4C–D, neither aging nor *Sirt1*^+^/^−^ sperm exhibited significant differences in the percentage of viable pY-positive cells or in overall pY levels relative to WT controls (WT vs *Sirt1*^+^/^−^ : *p* = 0.098; WT vs aged: *p* = 0.754; Old WT vs *Sirt1*^+^/^−^ : *p* = 0.147). However, when examining the spatial distribution of pY signals, *Sirt1*^+^/^−^ spermatozoa displayed a distinct pattern (Fig. 4E), characterized by a shift from a predominantly midpiece-enriched signal in WT males to a more pronounced presence in the principal piece (Fig. 4F). Although this observation does not allow mechanistic interpretation, it nevertheless reflects an altered pY pattern relative to WT sperm under identical capacitation conditions. Upon stimulation with progesterone, both aging and *Sirt1*^+^/^−^ sperm exhibited a reduced proportion of acrosome-reacted cells relative to WT counterparts (Fig. 4G), indicative of reduced capacitated status. As only fully capacitated spermatozoa can fertilize a mature oocyte, we assessed fertilization competence by IVF assays. Both aged and *Sirt1*^+^/^−^ cohorts exhibited a similarly reduced fertilization capacity compared to WT controls (Fig. 4I).

### Defective support of early embryogenesis by *Sirt1*^**+**^**/**^**−**^ and aged sperm reduces IVF outputs

**Fig. 5:**
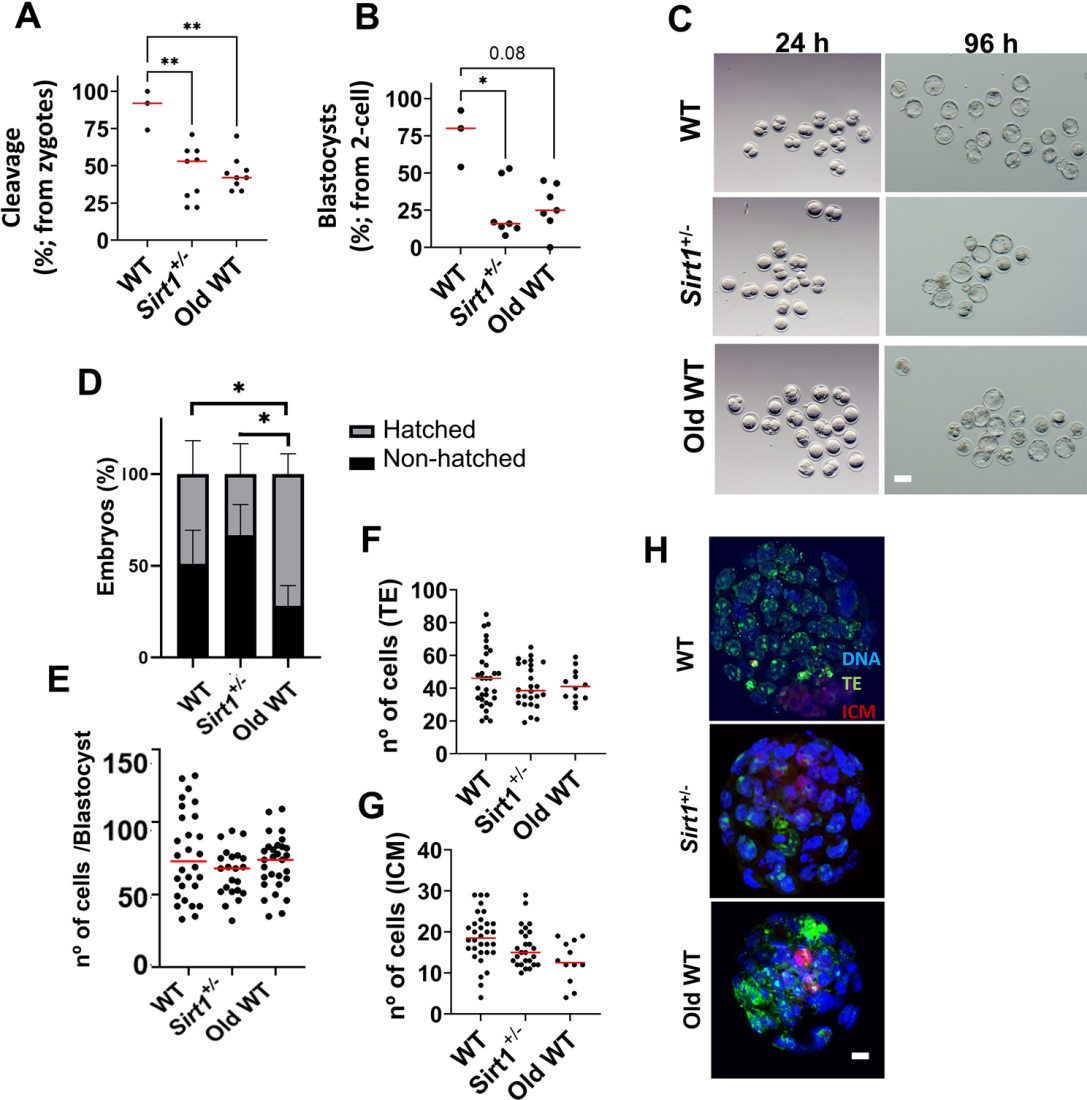
Testicular SIRT1 insufficiency and aging not only impair fertilization but also reduce the developmental potential of IVF-derived embryos. (**A**) the number of zygotes that cleaved to the 2-cell stage (**B**), and the proportion of 2-cell embryos that subsequently developed to the blastocyst stage, were quantified and expressed as percentages. (**C**) Representative pictures of 2-cell and blastocysts embryos. (**D**) proportion of hatched blastocysts derived from aged wt and *Sirt1*^+^/^−^ males in comparison to the wt controls. (**E**) total cell number per blastocyst and distribution of (**F**) trophectoderm (te) and (**G**) inner cell mass (ICM). (**H**) Representative immunofluorescence images of blastocysts stained for Cdx2 (green, te marker) and Oct4 (red, ICM marker), with DNA counterstained with DAPI (blue). Dots represent individual biological replicates for cleavage and blastocysts rates and for embryos for quality markers. Lines show median. Stacked columns represent mean ± s.e.m. (* *p*≤0.05, ** *p*≤0.01). Scale bar: 50 µm

Having established that fertilization competence is similarly compromised by SIRT1 insufficiency and aging, we next examined whether these spermatozoa could support early embryonic development. IVF assays revealed a pronounced reduction in cleavage rates in both cohorts relative to WT, with a significant decrease in the *Sirt1*^+^/^−^ group and a trend toward reduced blastocyst formation in the aged group (*p* = 0.08; Fig. 5A–B). This failure of zygotes to reach the 2-cell stage, and of 2-cell embryos to form blastocysts, indicates impaired early development. As all oocytes and maternal conditions were identical across groups, with comparable oocyte competence expected under these standardized settings, these outcomes suggest that sperm from both models may insufficiently support the earliest developmental transitions. Representative pictures of these observations are shown in Fig. 5C. Interestingly, while embryos derived from aged WT sperm displayed an increased hatching rate (Fig. 5D), further analyses indicated no significant differences in blastomere number or TE/ICM allocation among groups (Fig. 5E–G), suggesting that blastocyst quality remained largely preserved. Altered acTub levels were associated with impaired sperm function, including reduced mitochondrial activity, which is critical for fertilization. Given that suboptimal fertilization can influence cleavage timing and developmental competence [35, 36], we examined whether both characteristics showed any statistical association with the early embryo outcomes. Correlation analyses demonstrated that elevated acTub levels were negatively associated with early embryo development, whereas blastocyst quality parameters were unaffected (Table 2). In contrast, mitochondrial activity showed a strong positive correlation with 2-cell embryo and blastocyst formation, with no relation to blastocyst quality.

**Table 2:** Correlations among sperm mitochondrial activity and acTub with IVF outputs and blastocyst quality

Sperm	pY	AR	Cleavage	Blastocyst	Hatching	Blastomeres	TE	ICM
**Active mitochondria**	−0.146 *P* = 0.722	**0.753** *P*** = 0.024**	**0.767** *P*** = 0.021**	**0.683** *P=***0.05**	−0.254 *P* = 0.51	−0.017 *P* = 0.98	0.250 *P=*0.43	0.917 *P=*0.12
**acTub**	0.260 *P* = 0.515	**−0.698** *P*** = 0.045**	**−0.831** *P*** = 0.01**	**−0.712** *P*** = 0.04**	0.121 *P* = 0.77	0.153 *P* = 0.22	−0,525 *P=*0.15	−0,712 *P=*0.43

## Discussion

In this study, we show that SIRT1 haploinsufficiency modifies the testicular acetylome in a way that partially overlaps with aging, revealing shared alterations in selected transcription factors and proteins essential for sperm function. This altered profile coincides with the loss of proteins involved in cellular maintenance and stress protection during aging, together with the emergence of pro-senescence signatures (Iniesta-Cuerda et al., manuscript under final revision). Notably, we identified altered acetylation of ZNF638 and MORC4, previously unrecognized transcriptional regulators affected by reduced SIRT1 activity, whose modification may contribute to the transcriptional shifts reported in aging testes [37–39]. This expands the paradigm established for well-known SIRT1-dependent regulators such as p53, PGC-1α, and FOXO1/3 [14, 21, 40, 41], reinforcing the notion that SIRT1 plays a specific, non-redundant role in maintaining testicular homeostasis. Beyond these transcriptional regulators, the broader testicular acetylome displayed coordinated alterations across entire protein modules. A subset of acetylated proteins present only in WT testes was absent in *Sirt1*^+^/^−^ and aged samples, mapping to coherent functional categories: testicular structure (MFAP2) [42, 43], meiotic control (ANAPC7, PDS5B, ESCO1, CENPE, TOP1, THOC2, TPR) [44–47], flagellar organization and motility (CEP126, CEP170, CEP350, Anillin, AKAP13, CMYA5) [48–52] and signaling/metabolism (SHIP1, FERM2, GAK, ABCD4) [53–56]. Although SIRT1 is a deacetylase, the predominance of hypoacetylated proteins in both *Sirt1*^+^/^−^ and aged testes is compatible with the idea that global acetylation levels reflect the combined input of multiple regulatory processes, including HAT activity [57], pH-dependent modulation of the acetylome [58], and metabolic determinants such as acetyl-CoA availability [59]. These factors may reduce acetylation in specific protein subsets independently of SIRT1 levels, offering a plausible explanation for the mixed hyper- and hypoacetylation pattern observed. In line with these modules, several proteins differentially acetylated in *Sirt1*^+^/^−^ testes have documented knockout phenotypes affecting tubulin acetylation, flagellar organization, or midpiece integrity (e.g., CFAP58, Rootletin, CEP170). Such phenotypes implicate pathways that are directly relevant to sperm architecture and stability, providing functional context for interpreting the physiological alterations detected in mature spermatozoa [27].

Indeed, when extended to mature-state spermatozoa and we deep in their physiology, the acetylation imbalance is maintained, with α-tubulin hyperacetylation emerging as a key feature linking SIRT1 insufficiency and aging. Within this framework, CFAP58 appears as a structural component likely connecting a testis-imprinted acetylation program with the organization of the sperm midpiece. Confined to this region, CFAP58 has been implicated in supporting appropriate levels and spatial distribution of axonemal α-tubulin acetylation during spermiogenesis [60]. In *Sirt1*^+^/^−^ and aged testes, its aberrant acetylation likely compromises this supportive role and imprints a defect that later manifests as midpiece-restricted α-tubulin hyperacetylation, biasing axonemal microtubules toward constitutive stabilization. Consistent with this interpretation, loss of CFAP58 function in mice reduces α-tubulin acetylation along the sperm flagellum [60], and although K40-tubulin acetylation generally supports microtubule stability and motility [61], excessive or mislocalized acetylation, particularly within the midpiece, can be detrimental, constraining mitochondrial dynamics and energy homeostasis [62, 63]. Consistent with this view, sperm from *Sirt1*^+^/^−^ and aged males exhibited pronounced mitochondrial impairment, with acTub levels correlating negatively with mitochondrial activity, acrosomal responsiveness, and membrane integrity. Accordingly, midpiece-specific tubulin hyperacetylation could reflect a basal lesion in *Sirt1*^+^/^−^ and aged sperm, associated with mitochondrial dysfunction and with a flagellar rigidity that may compromise the transition into the curved, pliable, and flexible trajectories characteristic of hyperactivated motility, as previously observed under testicular SIRT1 insufficiency [27]. Thus, the acetylation imbalance due to age-related decline or genetically imposed SIRT1 insufficiency might not be merely structural but appears to be associated with functional constraints that persist in the mature sperm.

With this basal defect in place, the question arises as to whether these sperm are capable of developing capacitation and achieving fertilization effectively. Following 1 h under capacitating conditions, fewer *Sirt1*^+^/^−^ and aged sperm remained viable, and the survivors retained elevated acTub levels, providing a window for functional interrogation. Although these data do not allow mechanistic inference, the altered distribution of pY suggests that capacitation-associated signaling is initiated but does not follow the spatial dynamics typically observed in WT sperm. Such deviations point to disrupted coordination of signaling along the flagellum, consistent with previously described capacitation defects in this mode [28]. This raises the prospect that spatial precision, rather than overall pY amplitude alone, represents a sensitive aspect of capacitation affected in *Sirt1*^+^/^−^ males [34]. This altered topology is also compatible with deficiencies in cAMP/PKA-axis components required for capacitation and hyperactivation, specifically the absence of PKA-Cα and the sperm motility kinase SMOK in *Sirt1*^+^/^−^ and aged WT sperm (Iniesta-Cuerda et al., manuscript under final revision). As sperm-specific PKA-Cα2 orchestrates cAMP signaling essential for male fertility [64], its loss could underlie why pY magnitude appears preserved while its spatial progression is altered. Human data further support that efficient capacitation depends not only on PKA activation but also on the suppression of select Ser/Thr phosphatases [65], highlighting that proper routing of signals, rather than absolute signal strength, is essential for achieving capacitation. Functionally, progesterone-induced AR was reduced in both models, a phenotype consistent with two major constraints: impaired mitochondrial function (ATP supply, Ca^2 +^ handling) and excessive midpiece stabilization through hyperacetylated α-tubulin, both expected to limit hyperactivation [27] and exocytotic competence (herein). In this line, PLC-η1, a phosphoinositide-specific PLC involved in Ca^2 +^ signaling [66, 67], appears aberrantly acetylated in *Sirt1*^+^/^−^ and aged testes. Although we cannot infer its functional impact from the present data, such modification could plausibly influence Ca^2 +^ -dependent processes during germ-cell development. Within this working model, altered PLC-η1 acetylation may contribute to a cellular context that later becomes relevant at capacitation and AR, stages in which Ca^2 +^ –mitochondrial coupling is known to be rate-determining [68]. In agreement, our correlation analyses indicated that mitochondrial activity showed a stronger association with AR efficiency than bulk pY levels.

The downstream consequences of SIRT1 insufficiency extend into embryogenesis, becoming evident during the preimplantation development of *Sirt1*^+^/^−^ and aged-derived embryos. IVF assays revealed a reduced ability of these zygotes to progress to the 2-cell stage and, subsequently, to reach the blastocyst stage, with a trend toward diminished blastocyst formation, particularly in the aged group, while blastocyst quality parameters remained largely preserved. Notably, in our companion study of natural fertilization, both *Sirt1*^+^/^−^ and aged males showed reduced ability to sire pregnancies, and in vivo–derived embryos displayed lower progression to 2-cell and blastocyst stages, with *Sirt1*^+^/^−^ blastocysts also showing reduced quality (Iniesta-Cuerda et al., manuscript under final revision). Importantly, aged WT males carry two intact *Sirt1* alleles and therefore do not generate embryos with haploinsufficiency, yet they exhibit developmental defects that closely resemble those observed with *Sirt1*^+^/^−^ sperm. This parallel between a physiological model of aging and a genetic model of reduced SIRT1 activity suggests that the early developmental impairments may arise primarily from testis-derived sperm deficiencies rather than from the embryonic genotype. Within this framework, the fact that the IVF developed herein does not fully restore developmental progression observed in vivo is consistent with the possibility that embryos fertilized by functionally compromised sperm remain vulnerable despite assisted fertilization, further supporting a paternal, sperm-related contribution to these early developmental limitations. These findings raise the possibility that, beyond the structural and metabolic deficits characterized here, a lack of adequate epigenetic support, consistent with SIRT1’s established roles in chromatin regulation [69], may underline the impaired developmental potential of such embryos. Addressing this dimension is the focus of our forthcoming study, which seeks to determine how paternal SIRT1 insufficiency reshapes the embryonic epigenetic landscape.

## Conclusion

Together, our findings identify α-tubulin hyperacetylation in the sperm midpiece may represent a shared feature of both SIRT1 insufficiency and aging, with direct consequences for mitochondrial function, capacitation, and fertilization efficiency. This persistent modification supports a model in which loss of SIRT1 activity alters the acetylation of testicular proteins such as CFAP58, thereby propagating structural and metabolic defects to the mature sperm midpiece. By integrating proteomic, acetylomic, and functional data in a model of partial SIRT1 insufficiency that mirrors physiological aging and comparing it with naturally aged males, our study adds a distinct perspective by linking testicular and sperm acetylomes to sperm performance, capacitation, and fertilization outcomes.

## Electronic supplementary material

Below is the link to the electronic supplementary material.

Supplementary material 1

Supplementary material 2

## Data Availability

The acetylome datasets obtained from testicular tissue via LC–MS, supporting the characterization of the acetylation landscape in WT, *Sirt1*^+^/^−^, and aged WT testes, are provided in the Supplementary Data S2. All other data generated or analyzed during this study are included within the manuscript.
