# Supplementary material for: SIRT1 haploinsufficiency recapitulates age-associated subfertility through sperm α-tubulin hyperacetylation
Source: Biol Direct. 2026 Mar 25;21:45. doi: 10.1186/s13062-025-00710-2 (PMC13067630; doi:10.1186/s13062-025-00710-2)
Supplement: Supplementary file 1 — Supplementary material 1 [file 13062_2025_710_MOESM1_ESM.docx]

**Supplementary methods**

**Electrophoresis and Western Blot**

Both sperm and testes were lysed in RIPA buffer enriched with a complete mini protease inhibitor cocktail (Roche, Switzerland). Samples of sperm heads were prepared by sonication and lysis in radioimmunoprecipitation assay buffer supplemented with 100 mM DTT. Protein concentration was measured using the bicinchoninic acid method (Lovrien and Matulis, 2005; Olson and Markwell, 2016). Thereafter, the samples were mixed with Laemmli loading buffer supplemented with β-mercaptoethanol and loaded to 4-15% separating Mini-PROTEAN TGX Stain-Free^TM^ Precast Gels (Bio-Rad, France). Separated proteins were blotted using the Trans-Blot Turbo^TM^ Transfer System (Bio-Rad, France) onto PVDF membrane. The membrane was blocked in TBS added with Tween- 20 (T-TBS; 0.5% (v/v)) and BSA (5% (w/v) for 60 min at RT, and incubated with antibodies (1:1,000) overnight at 4°C as follows: anti-Acetyl Lysine (acLys; ADI-KAP-TF120-E, Enzo Life Sciences), anti-acetyl αTubulin (acTub; ab24610, Abcam, UK), and anti-αTubulin (#2144, Cell Signaling Technology). Horseradish peroxidase- conjugated secondary antibodies (goat anti-IgG; 1:15,000) were applied for 1 h at RT. The targeted proteins were visualized using ECL Select Western blotting Detection Reagent (GE Healthcare Life Sciences, United Kingdom), and membranes were scanned on a ChemiDocTM MP System (Bio-Rad, France). Images of membranes were processed using ImageLab 6.0.1 software (Bio- Rad, France) and the ’adjusted volume’ of the bands were recorded.

**Proteome profiling of testes by LC-MS**

Mouse testicular lysates in volume about 20 µL in RIPA buffer containing 100 µg of proteins were digested by trypsin according to a FASP protocol (Wiśniewski, Zougman, Nagaraj, & Mann, 2009), with the exception of alkylating agent where 100mM acrylamide was used. All chemicals needed for in-solution digestion were purchased from Sigma-Aldrich. Digests were pipetted out to 0.22 µm PVDF filter (OD 4 mm) and directly filtered to HPLC vial. Samples were analyzed on nanoLC system (nanoElute, Bruker Daltonics) coupled to a trapped ion mobility quadrupole time-of-flight mass spectrometer (timsTOF Pro, Bruker Daltonics) via a nanoelectrospray ion source (Captive Spray Source, Bruker Daltonics). Peptides (200-300 ng) were directly loaded and separated on an analytical column (100 mm × 75 μm, C18 ReproSil AQ, 1.9 μm) (Bruker TEN column, Bruker Daltonics). Peptides were eluted using 0.1% formic acid as mobile phase A at a flow rate of 300 nL/min and 30 minutes long gradient with liner increase of acetonitrile to 35% (the mobile phase B was ACN/0.1% formic acid) at a 40 °C column oven temperature. The eluting peptides were interrogated by an MS acquisition method recording spectra from 100 to 1700 m/z and ion mobility scanned from 0.6 to 1.6 Vs/cm^2^. The method consisted of a TIMS survey scan of 150 ms followed by six PASEF MS/MS scans, each 150 ms for ion accumulation and ramp time. The total cycle time was 0.5 s. The target intensity was 40,000, the intensity threshold was 1000, and singly charged peptides with m/z < 800 were excluded by an inclusion/exclusion polygon filter applied within the ion mobility over m/z heatmaps. Precursors for data-dependent acquisition were fragmented with an ion mobility-dependent collision energy, which was linearly increased from 20 to 59 eV. Data analysis: raw files were converted to mgf format using MSconvert version v3.0.22228 from ProteoWizard and proteins were identified using MASCOT search engine v2.7.0 (Matrix Science, UK). MS/MS spectra were searched against the Mouse Uniprot database (actualized 2023_01), setting the taxonomy to *Mouse musculus* (86411 sequences). Tandem MS data were searched with the following parameters: precursor mass tolerance of 20 ppm, fragment tolerance of 0.05 Da, trypsin specificity with a maximum of 2 missed cleavages, cysteine propionamide set as fixed modification, methionine oxidation, lysine acetylation and N-terminal acetylation as variable modification. False discovery rate (FDR) was estimated by running the searches against a randomized decoy database. Results of the identification step were filtered to proteins with FDR below 1%.

All samples from the three experimental groups (three biological replicates each) were processed and analysed in four technical replicates under identical conditions. Only those proteins whose acetylated peptides were consistently detected in all three biological replicates and in at least three technical replicates were retained for downstream analysis. Results of the retrieving are depicted in Venn diagram **Figure 4B** for whole proteome. Quantitative analysis was based on normalized emPAI values obtained from MASCOT search engine. Normalized emPAI values was imported into MarkerView (v1.2.1.1) for data normalization and unsupervised PCA analysis (weighting: logarithm, scaling: Pareto). Because the aim of this LC–MS dataset was restricted to a qualitative identification of acetylated peptides rather than quantitative differential analysis, no abundance-based contrasts (e.g., FDR-filtered fold changes, volcano plots, PCA clustering) were performed.
